# Quantitative assessment of the immune microenvironment in African American Triple Negative Breast Cancer: a case–control study

**DOI:** 10.1186/s13058-021-01493-w

**Published:** 2021-12-14

**Authors:** Vesal Yaghoobi, Myrto Moutafi, Thazin Nwe Aung, Vasiliki Pelekanou, Sanam Yaghoubi, Kim Blenman, Eiman Ibrahim, Ioannis A. Vathiotis, Saba Shafi, Anup Sharma, Tess O’Meara, Aileen I. Fernandez, Lajos Pusztai, David L. Rimm

**Affiliations:** 1 Department of Pathology, Yale University School of Medicine, 310 Cedar Street, BML 116, P.O. Box 208023, New Haven, CT 06520-8023 USA; 2 Genetics Branch, National Cancer Institute (NCI), National Institute of Health (NIH), Bethesda, MD USA; 3 Department of Internal Medicine, Section of Medical Oncology, Yale School of Medicine, New Haven, CT USA; 4 Yale Cancer Center, Yale School of Medicine, New Haven, CT USA; 5 Department of Surgery, Yale School of Medicine, New Haven, CT USA

**Keywords:** Triple-negative breast cancer, Tumor microenvironment, Quantitative immunofluorescence, African American, Immune cells

## Abstract

**Purpose:**

Triple negative breast cancer (TNBC) is more common in African American (AA) than Non-AA (NAA) population. We hypothesize that tumor microenvironment (TME) contributes to this disparity. Here, we use multiplex quantitative immunofluorescence to characterize the expression of immunologic biomarkers in the TME in both populations.

**Patients and methods:**

TNBC tumor resection specimen tissues from a 100-patient case: control cohort including 49 AA and 51 NAA were collected. TME markers including CD45, CD14, CD68, CD206, CD4, CD8, CD20, CD3, Ki67, GzB, Thy1, FAP, aSMA, CD34, Col4, VWF and PD-L1 we quantitatively assessed in every field of view. Mean expression levels were compared between cases and controls.

**Results:**

Although no significant differences were detected in individual lymphoid and myeloid markers, we found that infiltration with CD45^+^ immune cells (*p* = 0.0102) was higher in TNBC in AA population. AA TNBC tumors also had significantly higher level of lymphocytic infiltration defined as CD45^+^ CD14^−^ cells (*p* = 0.0081). CD3^+^ T-cells in AA tumors expressed significantly higher levels of Ki67 (0.0066) compared to NAAs, indicating that a higher percentage of AA tumors contained activated T-cells. All other biomarkers showed no significant differences between the AA and NAA group.

**Conclusions:**

While the TME in TNBC is rich in immune cells in both racial groups, there is a numerical increase in lymphoid infiltration in AA compared to NAA TNBC. Significantly, higher activated T cells seen in AA patients raises the possibility that there may be a subset of AA patients with improved response to immunotherapy.

**Supplementary Information:**

The online version contains supplementary material available at 10.1186/s13058-021-01493-w.

## Background

Solid tumor stroma is composed of many cell types including fibroblasts, immune cells (lymphocytes, macrophages, and myeloid derived stromal cells), and extracellular matrix (ECM). The cells and the signaling pathways in the tumor microenvironment (TME) resemble those associated wound healing suggesting that tumor stroma might be formed through abnormal activation of wound healing pathways [1–3]. The TME in breast cancer, especially the basal-like subtype, appears to modulate tumor behavior. Experimental studies using ionizing radiation suggest that certain processes in the TME such as high immune function favor the development of ER-negative tumors [4–7].

Younger age, breast remodeling after weaning, obesity, exposure to ionizing radiation, BRCA1 mutation and AA race are all factors that affect the immune microenvironment of tissues including that of breast tissue [8, 9]. The TME and secreted factors in breast tissue may be associated with the more basal-like breast tumors in these groups of patients [4–7].

The incidence rate of TNBC among AA women is approximately double that of Non-AA (NAA) women, with much of the disparity occurring at early ages [8, 10]. What accounts for this disparity is unclear; both reproductive factors (i.e., lower rate of breast feeding, multiple early pregnancies) and yet to be identified genetic predisposition to developing TNBC have been suggested [8, 11]. While the rate of germline BRCA1/2 mutations in patients with breast cancer is similar across racial/ ethnic groups; obesity, higher parity, younger age at first birth, and early weaning or never-having breast fed are more prevalent among AA than white women and may significantly contribute to increased inflammation and to higher TNBC incidence in AA women [9, 10]. This chronic low-grade inflammation is a complex immune response may play an important role in the pathogenesis of multiple chronic diseases, which are more prevalent among the AA population [9, 12–14]. Even though several pathways have been identified for chronic low-grade inflammation and candidate loci have been proposed [12], the contribution of sociodemographic factors and lifestyle to maintenance of such an inflammatory state has also been shown [15].

In summary, subtle differences in the immune TME exist between AA and NAA patients [7, 16]. Profibrotic conditions, including keloid scarring, uterine leiomyoma, the glomerulosclerotic conditions associated with end-stage renal disease and sarcoidosis are more commonly seen in AA patients [9]. The effect of this inherent immune dysregulation and the resultant inflammation on tissues, may predispose AA population to a higher prevalence of basal-like tumors [17]. Here, we propose the stromal cellular content may be an important factor modulating the higher rate of TNBC formation in AA women. By comparing TME in TNBCs of AA vs that of NAA patients we hoped to find specific immune inflammatory protein signatures associated with TNBCs that may assist in future classification and management of the disease. Understanding the relative contributions of immunological factors and their possible interactions is essential for to improve the survival and quality of life among AA women with TNBC.

## Methods

### Tissue collection and patient cohort

A total of 100 TNBC cases were reviewed and selected from the Yale University Pathology archives. Specifically, treatment-naïve cases were selected by the pathologist if there was an abundance of tumor present in the blocks for future study. Areas of invasive tumor were identified by a pathologist and circled on the whole tissue section, giving careful attention to avoid areas with admixed in situ and/or benign tissue. Patients’ demographic characteristics are described in Table 1. The Yale Human Investigation Committee approved the patient consent forms or in some cases a waiver of consent all in accordance with the ethical guidelines of the US Common Rule (protocol #9505008219).

**Table 1:** AA and Non-AA patient information

Characteristic	African American, N = 49^a^	Non-African American, N = 51^a^	*p* value^b^
Age	59 (47,66)	55 (49,66)	0.8
Ethnicity			0.3
Hispanic or Latino	1 (2%)	0(0%)	
Non-Hispanic	40 (82%)	38 (75%)	
Unknown	8 (16%)	13 (25%)	
BMI	31.0 (27.0, 35.3)	26.6 (24.0, 31.9)	0.009
Unknown	12	12	
Stage (AJCC 8th edition)			0.5
IA	17 (35%)	18 (35%)	
IIA	17 (35%)	19 (37%)	
IIB	13 (27%)	8 (16%)	
IIIA	2 (4.1%)	4 (7.8%)	
IIIB	0 (0%)	2 (3.9%)	
Grade			0.3
2	6 (12%)	10 (20%)	
3	43 (88%)	41 (80%)	
Histology			0.6
AdenoSquamous	1 (2.2%)	0 (0%)	
Fibromatoid nodule	1 (2.2%)	0 (0%)	
IDC	40 (87%)	44 (86%)	
IDC-medullary	1 (2.2%)	0 (0%)	
IDC-micropapillary	1 (2.2%)	3 (5.9%)	
ILC	0 (0%)	1 (2.0%)	
IMC	2 (4.3%)	1 (2.0%)	
Metaplastic	0 (0%)	1 (2.0%)	
Squamoid	0 (0%)	1 (2.0%)	
Unknown	3	0	
Chemotherapy	37 (76%)	38 (81%)	0.5
Unknown	0	7	
XRT	41 (84%)	31 (70%)	0.13
Unknown	0	7	
Follow up (years)	6.1 (2.9, 11.0)	5.4 (1.9, 10.2)	0.2
Unknown	1	0	

^a^Median (IQR); n (%)

^b^Wilcoxon rank sum test; Fisher’s exact test; Pearson’s Chi-squared test

### Experimental design

Characterization of T cell subsets in resected tissues was performed using multiplexed quantitative immunofluorescence. We used CD45 positivity as a marker of all immune cells, including lymphoid and myeloid cells. CD14 further distinguished myeloid from lymphoid cells. Among myeloid-derived cells we sought to identify macrophages (CD68^+^) and more specifically alternatively activated macrophages or CD206^+^ cells, which may alter the disease outcome [18].

Similarly, we investigated the different immune cell types that belong to lymphoid cells; T-cells (CD3^+^) and B-cells (CD20^+^). CD3^+^ T-cells, were subsequently, divided into helper T-cells (CD4^+^) and cytotoxic T-cells (CD8^+^). After phenotyping typical CD3^+^ cells, we selected activated CD3^+^ cells, as high expressors of Ki67 and Granzyme B (GZMB). We used median Automated quantitative analysis (AQUA) score as the cutoff to determine high versus low Ki67/ GZMB expression and active vs dormant CD3^+^ cells, respectively [19]. Regulatory T-cells (Tregs) were defined by CD4 and FOXP3 a double-positive phenotype.

In order to evaluate cancer-associated fibroblasts, we utilized different mesenchymal phenotype markers. Thy1 + FAP + a-SMA + phenotype represented stromal fibroblasts [20]. Since smooth muscle surrounds both breast ducts and endothelial cells, we used a CD34 + COL4 + vWF + phenotype to exclude these a-SMA + cell types that could skew our results.

Finally, we examined the level of expression of the Programmed Death-Ligand 1 (PD-L1) in tumor cells (Cytokeratin positive, CK^+^), stroma cells (CK^−^) and macrophages (CD68^+^). PD-L1 has a key role in tumor immune evasion and more specifically in TNBC, where incorporation of immunotherapy in the neoadjuvant setting and metastatic disease has been recently approved [21, 22].

### Antibodies, quantitative immunofluorescence (QIF)

#### Antibodies

Expression of CD45, CD14, CD8, CD20, CD68, CD206, PD-L1, THY1, FAP, CD3, Ki67, GZMB, CD4,FOXP3, a-SMA, CD34, COL4/vWF was evaluated using monoclonal antibodies as follows; for CD45: monoclonal mouse IgG1 antihuman 2B11 + PD7/26 (DAKO, Carpinteria, CA), for CD14: monoclonal rabbit antihuman D7A2T (Cell Signaling Technology/CST, Danvers, MA), for CD8: monoclonal mouse IgG1 antihuman C8/144B (DAKO, Carpinteria, CA), for CD20: monoclonal mouse IgG2a antihuman L26, for CD68: monoclonal mouse antihuman IgG3 PG-M1 (DAKO, Carpinteria, CA), for CD206: monoclonal rabbit antihuman EPR22489-7 (Abcam, Cambridge, UK), for PD-L1: monoclonal rabbit antihuman SP142 (Abcam, Cambridge, UK), for THY1: monoclonal mouse IgG1 7E1B11 (Abcam, Cambridge, UK), for FAP: monoclonal rabbit antihuman EPR20021 (Abcam, Cambridge, UK), for CD3: monoclonal rabbit antihuman SP7 (Littleton, CO), for Ki67: monoclonal mouse IgG1 antihuman MIB-1 (DAKO, Carpinteria, CA), for GZMB: monoclonal mouse IgG2a antihuman GZB01 (LSBio, Seattle, WA), for CD4: monoclonal rabbit antihuman EPR6855 (Abcam, Cambridge, UK), for FOXP3: monoclonal rabbit antihuman D2W8E (CST, Danvers, MA), for a-SMA: monoclonal mouse IgG2a antihuman 1A4 (DAKO, Carpinteria, CA), for CD34: monoclonal rabbit antihuman EP373Y (Abcam, Cambridge, UK), for COL4: monoclonal mouse IgG1 antihuman COL-94 (Abcam, Cambridge, UK), and for vWF: monoclonal mouse IgG1 antihuman F8/86 (DAKO, Carpinteria, CA).

Cytokeratin at 1:100 dilution (polyclonal rabbit anti-cow cytokeratin, wide spectrum screening, DAKO, Carpinteria, CA) or (monoclonal mouse IgG1 antihuman Clone AE1/AE3, DAKO Carpinteria, CA) was used to identify the tumor cells. Rabbit EnVision reagent (DAKO,neat), Goat Anti-Mouse IgG2a heavy chain (Horseradish peroxidase, HRP) (Abcam), Rat anti-Mouse IgG1 Secondary Antibody, HRP, eBioscience (ThermoFisher Scientific, Waltham, MA USA), Goat Anti-Mouse IgG3 heavy chain (Horseradish peroxidase, HRP) (Abcam), Alexa 546 conjugated goat antirabbit secondary antibody (Molecular Probes, Eugene, OR) were used as secondary antibodies. Tyramide-bound fluorophores were added after each secondary antibody to bind to the HRPs. Cyanine 5 (Cy5) directly conjugated to tyramide (Akoya, Waltham, MA, USA), Alexa Fluor 750 streptavidin (Cy7) (ThermoFisher Scientific, Waltham, MA USA) and Tyramide Signal Amplification (TSA) fluorescein (Fluorescein isothiocyanate (FITC)) (AKOYA, Marlborough, MA 01752, USA) were used for target antibody detection. Additional file 1 shows all the antibodies we used with the dilutions and stock concentrations.

#### Staining of whole tissue sections

Formalin-Fixed Paraffin-Embedded whole tissue sections were deparaffinized with xylene. Rehydration was performed using ethanol. Antigen retrieval was performed using EDTA buffer pH 8.0 at 97 °C for 20 min in a pressure-boiling container (PT Module, Lab Vision, Thermo Scientific, Waltham, MA). 2.5% hydroxyl peroxide in methanol for 30 min was used to block endogenous peroxidase activity, followed by 0.3% bovine serum albumin in 0.1 mol/L of Tris-buffered saline for 30 min at room temperature. This was followed by incubation of the slides with the primary antibodies and cytokeratin. Slides were then incubated at 1-h room temperature with the primary antibodies and with cytokeratin. Secondary antibodies and tyramide-bound fluorophores were used for target antibody detection. 4,6-diamidino-2-phenyl-indole (DAPI) was used to stain nuclei and mounted with Prolong Gold antifade mounting reagent (P36394, Life Technologies). All staining was performed using the Lab Vision Autostainer 720 (Thermo Scientific).

#### Fluorescence measurement and scoring

Quantitative immunofluorescence (QIF) was assessed using two platforms: image collection was performed on either the PM2000 (HistoRx) or Vectra Polaris (Perkin Elmer) automated fluorescence microscopy platforms. The final images were analyzed and quantified using the AQUAnalysis Software (Navigate Biopharma) or the InForm Software (Perkin Elmer), respectively.

Image analysis with AQUA method of QIF was used initially for all the markers CD45, CD14, CD8, CD20, CD68, CD206, PD-L1, THY1, FAP, CD3, Ki67, GZMB, CD4, FOXP3, a-SMA, CD34, COL4/vWF. A QIF score was generated by dividing the sum of target pixel intensities by the area of the molecularly designated compartment, as previously described. In order to distinguish tumor from tissue stroma and other components, an epithelial tumor “mask” was created by binarizing the cytokeratin signal and creating an epithelial compartment. Stromal “mask” is created by subtracting tumor mask from the nuclear mask and is representative of tumor microenvironment. The expression level of a maximum of 3 targets can be measured in the above molecular compartments.

For the whole sections, using a × 20 objective, a series of image fields of view (FOV) were captured within the circled invasive tumor to ultimately cover the tissue of interest. Depending on the size of the tumor, 40–200 fields were captured per section. QIF scores were normalized to the exposure time and bit depth at which the images were captured, to compensate for any variability. All acquired histospots were visually assessed and cases with staining artifacts or less than 2% tumor (cytokeratin staining) were omitted from the analysis. Since the expression of Tregs was low in our samples, we quantified Tregs, which are characterized by the expression of CD4 and FOXP3, using the InForm Software, as previously described [23]. Multispectral images were captured by Vectra Polaris in three channels on all tumor tissues. With the trainable feature-recognition InForm software, Tregs and tissue types were identified. Cell nuclei were visualized by the signal from DAPI stain; cytokeratin was visualized with Alexa 546 fluorophore; and the proteins of interest were visualized with the Cy5, Cy7 and FITC dye.

The cell count for phenotype of interest (CD4 and FOXP3 double-positive phenotype) was calculated in both tumor and stromal tissue for final analysis, using the segmentation software, InForm Tissue Finder (PerkinElmer, Waltham, MA, USA). The area of each tissue category, tumor and stroma, was evaluated to assess the density of Tregs, represented by (number of Tregs)/(pixel area) in each FOV.

#### Validation of staining and protein expression

Yale-Tissue microarray (YTMA) 405, a cell-line tissue microarray for immune cells, was used as positive control and for day-to-day standardization of assays. It revealed the expected levels and expression patterns of the immune cell biomarkers. YTMA 417, a random breast cancer tissue microarray was used as a second control. QIF scores for each marker, showed high concordance and remained reproducible, among control slides stained in different batches indicating limited inter-batch variation.

To show specificity and reproducibility, all antibodies were validated using an antibody validation protocol described previously [24]. Briefly, we have tested the antibodies in different conditions and chose the one that maximizes their dynamic range and the signal to noise ratio. Once the optimal conditions and concentrations have been determined, we proceeded with the tissue staining and further analysis.

### Statistical analysis

We used the median QIF scores of every marker from all FOV in each section to represent marker expression at the section level. Statistical comparisons between AA and NAA population were made using the Mann–Whitney U test except for comparison of T-cell status for which Fisher’s exact test was used. Statistical analyses were performed using the GraphPad Prism v6.0 for Windows (GraphPad Software, Inc, San Diego, CA, USA). Significance is defined as *p* < 0.05.

## Results

**Fig. 1:**
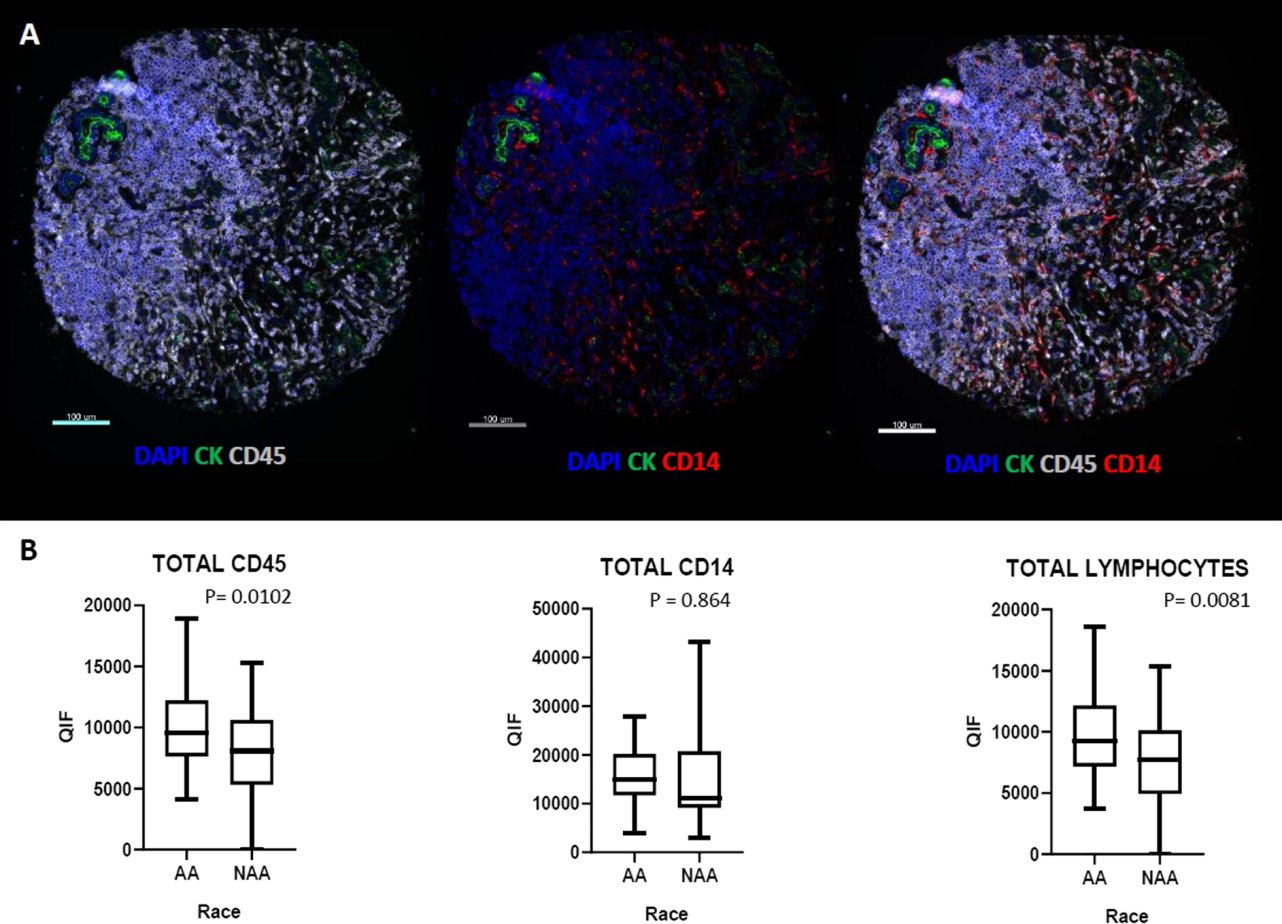
Comparison of pan-myeloid and pan-lymphoid immune cell in AA versus Non-AA TNBC tumors. **A** Representative images of multiplex immunofluorescent staining for CD45 and CD14 markers. **B** Comparing CD45^+^ (all immune cells), CD14^+^ (myeloid) and CD45^+^ CD14—(lymphoid) cells in AA versus Non-AA TNBC tumors

The AA patients with TNBC have been shown to be associated with increased inflammation in TNBC [25] and were assessed here by the general antibody for leukocytes (CD45 or leukocyte common antigen). We found that quantitative scoring showed that infiltration with CD45^+^ immune cells (*p* = 0.0102) is higher in TNBC in AA samples, including higher level of lymphoid, CD45^+^ CD14^−^ (*p* = 0.0081) and myeloid CD45^+^ CD14^+^ cells (*p* = 0.0864) (Fig. 1).

**Fig. 2:**
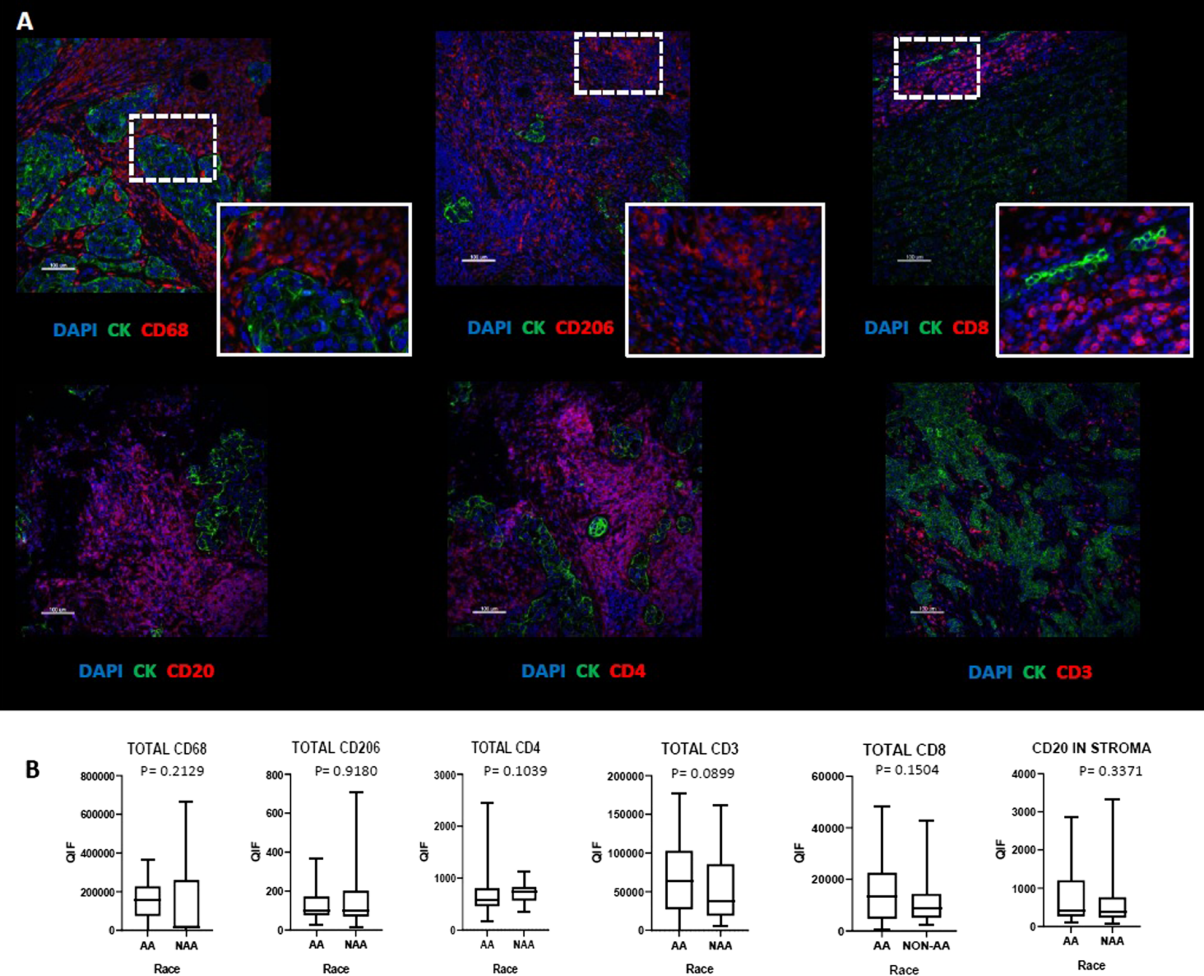
Comparison of myeloid and lymphoid subsets between AA and Non-AA TNBC tumors. **A** Representative images of multiplex immunofluorescent staining for two macrophage markers: CD68 and CD206, T cell markers: CD3, CD4 and CD8, and B-cell marker: CD20. **B** Comparison of the level of the above markers in AA versus Non-AA TNBC tumors

Quantification of expression of CD68, CD206, CD4, CD83, CD8, CD20 assessed the specific lymphoid and myeloid components of the TNBC microenvironment. Figure 2 illustrates the range of expression of the lymphoid and myeloid cell subtypes between AA and NAA patients. No significant differences were observed among individual immune cell subtypes, although CD3^+^, CD8^+^, and CD20^+^ cells were, on average, numerically higher among AA TNBC samples.

**Fig. 3:**
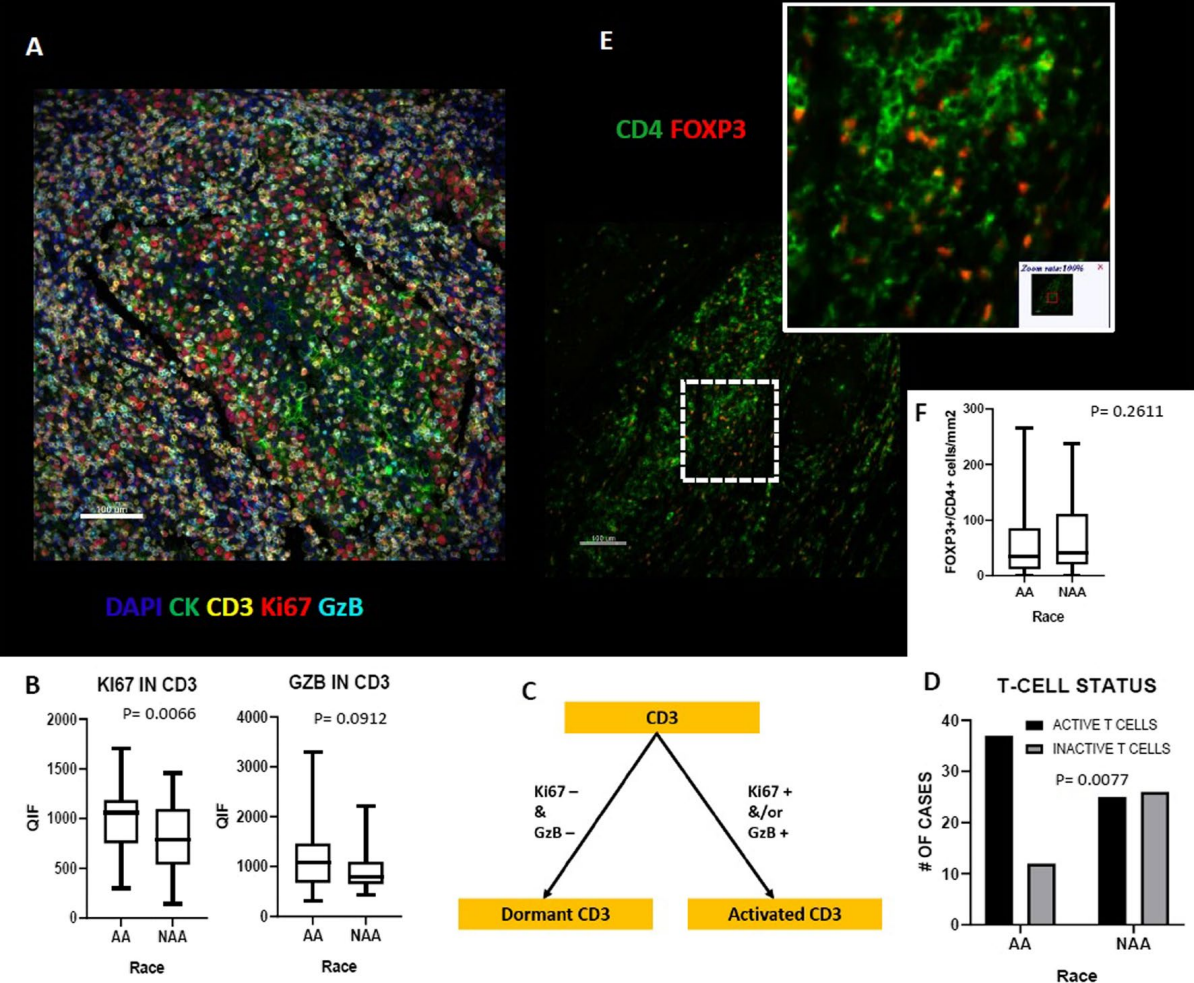
Comparison of T-cells status and Regulatory T-Cell population in AA versus Non-AA TNBC. **A** Representative image of multiplex immunofluorescent staining for CD3, Ki67 and GzB markers **B** Comparison of Ki67 and GzB in T-cells of AA vs Non-AA TNBC tumors. **C** Schema of activation status of T cells. **D** Comparison of the activation status of T-Cell in AA vs Non-AA TNBC tumors. **E** Representative image of multiplex immunofluorescent staining for CD4 and FOXP3 markers. **F** Comparison of regulatory T-cell population in AA versus Non-AA TNBC tumors

We used a definition of activated T-cells as reported previously by Gettinger et al. [19], which showed association with response to immunotherapy in lung cancer. CD3^+^ cells of AA TNBCs were more likely to be in active than dormant state (*p* = 0.0077), compared to NAA TNBCs. Figure 3 shows a schematic for the definition of activated T cells and suggests a more active immunosurveillance in the AA TME. We observed significantly higher Ki67 expression in AA TNBCs (*p* = 0.0066); GzB level was also higher in AA TNBC (*p* = 0.0912), but the difference did not reach the statistical significance. No significant differences were detected in the level of regulatory T-cells (FOXP3^+^ CD4^+^) between AA and NAA tumors.

**Fig. 4:**
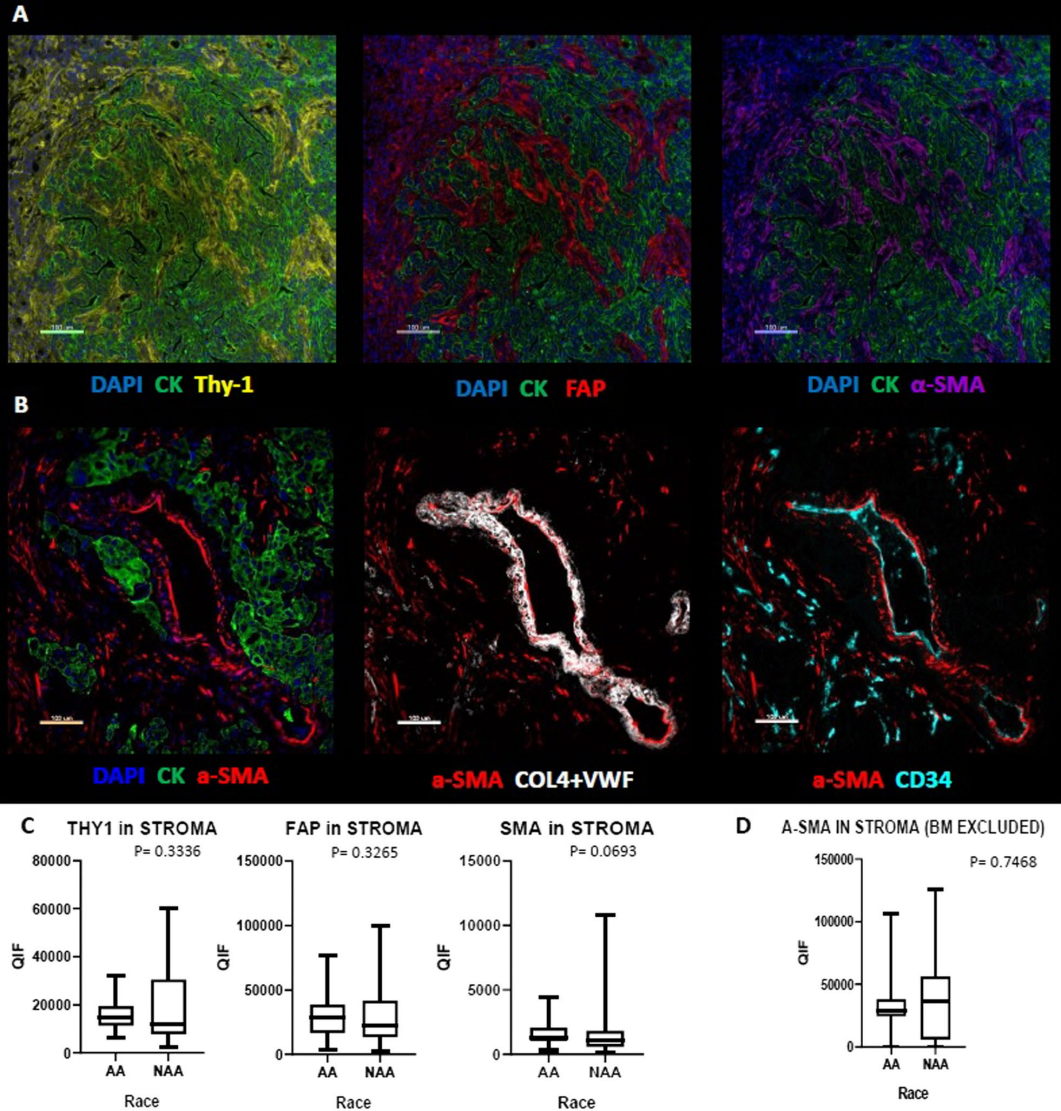
Comparison of Fibroblast markers in AA versus Non-AA TNBC tumors. **A** Representative images of multiplex immunofluorescent staining for Thy1, FAP and a-SMA markers. **B** Representative images of multiplex immunofluorescent staining for a-SMA, Col4-VWF and CD34. **C** Comparison of Thy-1-, FAP- and a-SMA- positive cells in AA versus Non-AA TNBC tumors. **D** Comparison of non-basement membrane a-SMA (a-SMA in Stroma—BM) in AA versus Non-AA TNBC tumors (Col4-VWF and CD34 masks have been used to eliminate basement membrane associated a-SMA from myofibroblastic a-SMA)

There is a well described increased propensity for keloid in AA compared to NAA populations (AAs are 20 times more likely to develop keloid than NAA individuals) [9]; This finding led us to hypothesize that the reaction to tissue injury would be expressed as a higher level of fibroblastic markers in the tumors of AA patients. However, none of the biomarkers we tested related to fibrous proliferation showed any significant difference between AA and NAA TNBC cases. Although the level of a-SMA was numerically higher in AA TNBC, the difference was not statistically significant and when the vascular and glandular a-SMA were manually excluded from the analysis the difference was lost (Fig. 4).

**Fig. 5:**
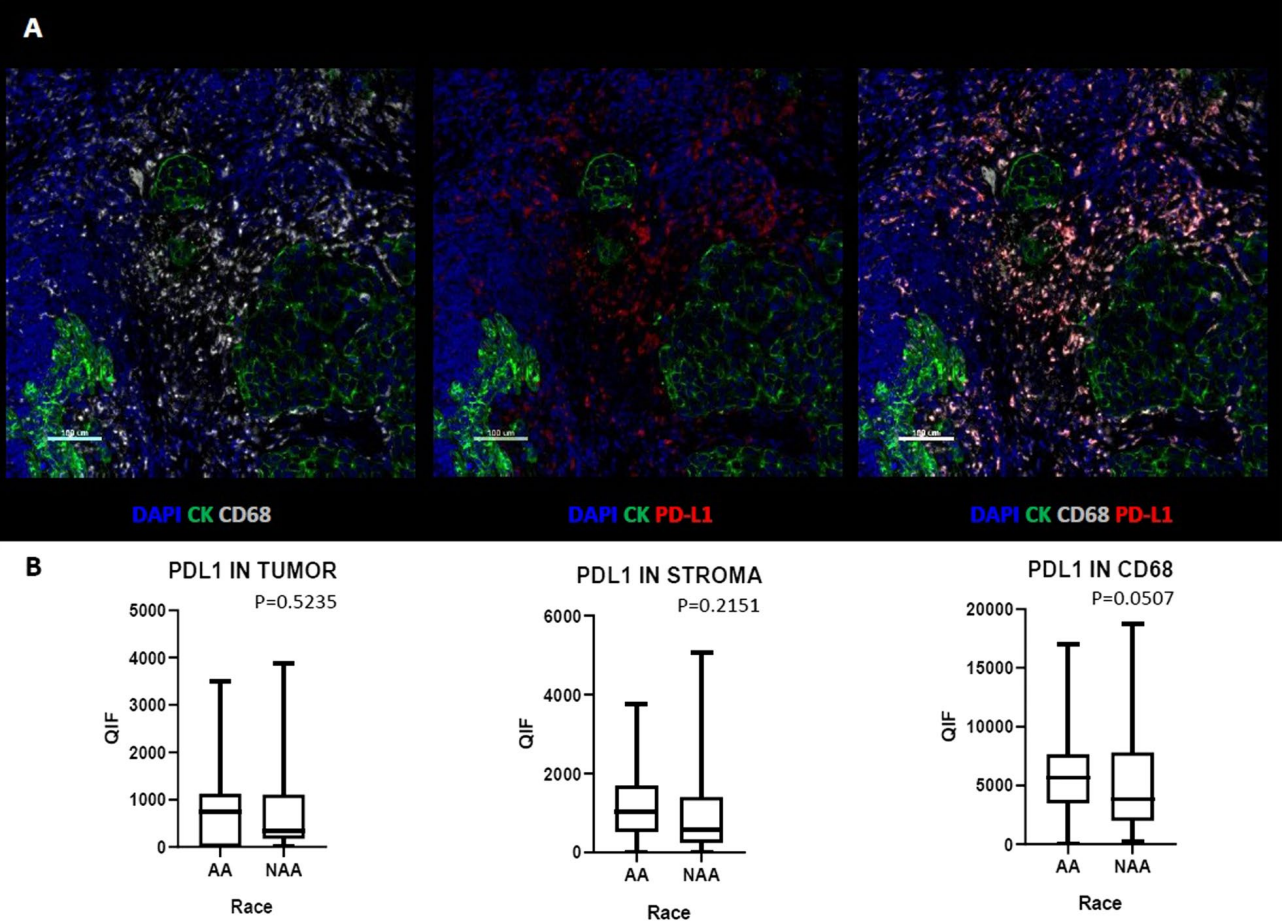
Comparison of PD-L1 marker in AA versus Non-AA TNBC tumors. **A** Representative images of multiplex immunofluorescent staining for CD68 and PD-L1 markers. **B** Comparison of PD-L1 expression in AA versus Non-AA TNBC tumors

In addition, we assessed PD-L1 expression in tumor, stroma and in macrophages (CD68^+^). We observed a marginally significantly higher expression of PD-L1 in macrophages (*p* = 0.0507) in AA TNBC cases compared with NAA but otherwise levels of PD-L1 are equivalent (Fig. 5).

## Discussion

Compared with NAA women, AA women have lower age-adjusted incidence rates of breast cancer diagnoses. However, the age-adjusted breast cancer mortality rates are higher in AA women. Racial differences remain after adjustment for sociodemographic and pathologic variables [26]. TNBC, which has the worse prognosis compared to other breast cancer subtypes [27], account for a higher percentage of breast cancer cases in AA than European American women [6, 27, 28]. The worse prognosis of breast cancer in AA women has partially been attributed to the higher prevalence of TNBC in this group. However, no survival difference for TNBC was detected between the two population [29].

We hypothesized that the breast stromal environment might be responsible for the higher frequency of Triple Negative Breast Cancer (TNBC) in African American (AA) vs White American women. We used quantitative immunohistochemistry as our approach to look for any evidence of differences in TME of TNBC between the two populations focusing on cells that modulate the immune response. We showed that differences exist in TME of TNBC between the two populations including that TNBC in AA women have a significantly higher level of lymphoid cells (CD45^+^ CD14^−^). However, we did not see any differences in the level of myeloid markers between the two populations. When compared different lymphoid markers between the two population, AA tumors had a numerically higher level of CD3, CD8, and CD20 markers but none of these differences were statistically significant. We found T-cells in AA tumors show higher levels of proliferation and compared to White Americans, an increase in activated T-cells (defined by higher levels of Ki-67 and/or GzB).

Although several studies have indicated that racial disparity in breast cancer outcome between patients of African compared to those of Caucasian ancestry are due to biological factors including differences in gene expression patterns of tumor cells as well as differences in tumor microenvironment (TME) between the two populations [26, 30–51], to our knowledge our study is the first one that have implemented quantitative immunofluorescence technique to compare the cellular contents of the TME of TNBC between AA and NAA American tumors. A recent study showed a stronger overall immune response in Black women compared with NAA women and those immune reactions shift the TME toward humoral immunity with a higher proportion of the T-cell exhaustion in the TME. This study demonstrated the most statistically significant and consistent racial differences in immune infiltrates were higher fractions of CD4^+^ T cells in samples from AA patients compared with NAA patients [52]. Results from a prior TCGA analysis also showed African ancestry to be associated with a higher leukocyte fraction in breast tumors [52, 53]. Racial disparities in TME have also been shown to exist in other types of cancers such as colon and prostate tumors [54–56].

Significantly higher levels of CD45^+^ CD14^−^ cells and the increased number of tumors with activated T-cells among the samples from African American patients are consistent with the concept of African American TME having a higher inflammatory response to tissue injury compared to White American TME. While we found CD3 was higher in the tumors, this difference did not reach the statistical significance level. However, the significantly higher BMI in the AA group might also be responsible for the higher breast inflammation of this group. This higher inflammation, to some extent, might be responsible for the significantly higher ratio of TNBC to other breast cancer subtypes in the AA population [57].

## Conclusion

Our study has a number of limitations. While we hypothesized differences in the stromal components associated with race, we assessed only the immune cell component of the stroma. ECM proteins and glycoproteins are major components of microenvironment that we were unable to assess. While we examine lymphoid, myeloid cells and fibroblasts, the extracellular stromal elements are challenging to measure with quantitative immunofluorescence methods. Furthermore, while the case control format allowed discovery of a few differences between AA and NAA TNBC, it is possible that more cases and controls would have pushed our numerical differences to statistical significance. In summary, we show that lymphoid cells are more prominently expressed in the TME in AA TNBC than NAA TNBC, but this finding is not seen for myeloid cells.

## Supplementary Information

**Additional file 1**. Primary antibodies stock concentrations and dilutions. ^1^Stock concentration not available

## Data Availability

The datasets generated and/or analyzed during the current study are not publicly available due the complexity of image analysis data, but are data is available from the corresponding author on reasonable request.

## Abbreviations

TNBC: Triple Negative Breast Cancer
AA: African American
NAA: Non-African American
QIF: Quantitative immunofluorescence
BMI: Body mass index
TME: Tumor microenvironment
ECM: Extracellular matrix
PD-L1: Programmed Death-Ligand 1
AQUA: Automated Death-Ligand
FOV: Fields of view

## References

[1] Harbeck N (2019). Breast cancer. Nat Rev Dis Primers.

[2] Dvorak HF (1986). Tumors: wounds that do not heal. Similarities between tumor stroma generation and wound healing. N Engl J Med.

[3] Foster DS (2018). The evolving relationship of wound healing and tumor stroma. JCI Insight.

[4] Roswall P (2018). Microenvironmental control of breast cancer subtype elicited through paracrine platelet-derived growth factor-CC signaling. Nat Med.

[5] Quail DF, Joyce JA (2013). Microenvironmental regulation of tumor progression and metastasis. Nat Med.

[6] Barcellos-Hoff MH (2013). Does microenvironment contribute to the etiology of estrogen receptor-negative breast cancer?. Clin Cancer Res.

[7] Hong CC (2013). Pretreatment levels of circulating Th1 and Th2 cytokines, and their ratios, are associated with ER-negative and triple negative breast cancers. Breast Cancer Res Treat.

[8] Ogony JW (2020). Immune responses and risk of triple-negative breast cancer: implications for higher rates among African American women. Cancer Prev Res (Phila).

[9] Byun JS (2018). Linking Race, Cancer Outcomes, And Tissue Repair. Am J Pathol.

[10] Zavala VA (2021). Cancer health disparities in racial/ethnic minorities in the United States. Br J Cancer.

[11] Shinde SS (2010). Higher parity and shorter breastfeeding duration: association with triple-negative phenotype of breast cancer. Cancer.

[12] Ligthart S (2016). DNA methylation signatures of chronic low-grade inflammation are associated with complex diseases. Genome Biol.

[13] Kovesdy CP (2018). CKD in African Americans as a complex intertwining of biology and socioeconomics: an introduction. Am J Kidney Dis.

[14] Feairheller DL (2011). Racial differences in oxidative stress and inflammation: in vitro and in vivo. Clin Transl Sci.

[15] Koster A (2006). Association of inflammatory markers with socioeconomic status. J Gerontol A Biol Sci Med Sci.

[16] Paalani M (2011). Determinants of inflammatory markers in a bi-ethnic population. Ethn Dis.

[17] Michael S (2016). Inflammation shapes stem cells and stemness during infection and beyond. Front Cell Dev Biol.

[18] Ch’ng ES, Jaafar H, Tuan Sharif SE (2011). Breast tumor angiogenesis and tumor-associated macrophages: histopathologist’s perspective. Patholog Res Int.

[19] Gettinger SN (2018). A dormant TIL phenotype defines non-small cell lung carcinomas sensitive to immune checkpoint blockers. Nat Commun.

[20] Wong PF (2019). Multiplex quantitative analysis of cancer-associated fibroblasts and immunotherapy outcome in metastatic melanoma. J Immunother Cancer.

[21] Schmid P (2020). Pembrolizumab for early triple-negative breast cancer. N Engl J Med.

[22] Schmid P (2018). Atezolizumab and nab-paclitaxel in advanced triple-negative breast cancer. N Engl J Med.

[23] Camp RL, Chung GG, Rimm DL (2002). Automated subcellular localization and quantification of protein expression in tissue microarrays. Nat Med.

[24] MacNeil T (2020). Antibody validation for protein expression on tissue slides: a protocol for immunohistochemistry. Biotechniques.

[25] O'Meara T (2019). Immune microenvironment of triple-negative breast cancer in African-American and Caucasian women. Breast Cancer Res Treat.

[26] Dietze EC (2015). Triple-negative breast cancer in African-American women: disparities versus biology. Nat Rev Cancer.

[27] Ihemelandu CU (2007). Molecular breast cancer subtypes in premenopausal and postmenopausal African-American women: age-specific prevalence and survival. J Surg Res.

[28] Kong X (2020). Variation in breast cancer subtype incidence and distribution by race/ethnicity in the United States from 2010 to 2015. JAMA Netw Open.

[29] Warner ET (2015). Racial and ethnic differences in breast cancer survival: mediating effect of tumor characteristics and sociodemographic and treatment factors. J Clin Oncol.

[30] Kim G (2020). The contribution of race to breast tumor microenvironment composition and disease progression. Front Oncol.

[31] Storz P (2005). Reactive oxygen species in tumor progression. Front Biosci.

[32] Martin DN (2009). Differences in the tumor microenvironment between African-American and European-American breast cancer patients. PLoS ONE.

[33] Koru-Sengul T (2016). Breast cancers from black women exhibit higher numbers of immunosuppressive macrophages with proliferative activity and of crown-like structures associated with lower survival compared to non-black Latinas and Caucasians. Breast Cancer Res Treat.

[34] Lazarus R (2002). Single nucleotide polymorphisms in innate immunity genes: abundant variation and potential role in complex human disease. Immunol Rev.

[35] Deshmukh SK (2015). Resistin and interleukin-6 exhibit racially-disparate expression in breast cancer patients, display molecular association and promote growth and aggressiveness of tumor cells through STAT3 activation. Oncotarget.

[36] Park NJ, Kang DH (2013). Inflammatory cytokine levels and breast cancer risk factors: racial differences of healthy Caucasian and African American women. Oncol Nurs Forum.

[37] Stewart PA (2013). Differentially expressed transcripts and dysregulated signaling pathways and networks in African American breast cancer. PLoS ONE.

[38] Mukhtar RA (2011). Elevated PCNA+ tumor-associated macrophages in breast cancer are associated with early recurrence and non-Caucasian ethnicity. Breast Cancer Res Treat.

[39] Dookeran KA (2010). p53 as a marker of prognosis in African-American women with breast cancer. Ann Surg Oncol.

[40] Mehrotra J (2004). Estrogen receptor/progesterone receptor-negative breast cancers of young African-American women have a higher frequency of methylation of multiple genes than those of Caucasian women. Clin Cancer Res.

[41] Ademuyiwa FO (2017). Differences in the mutational landscape of triple-negative breast cancer in African Americans and Caucasians. Breast Cancer Res Treat.

[42] Kalla Singh S (2010). Insulin-like growth factors I and II receptors in the breast cancer survival disparity among African-American women. Growth Horm IGF Res.

[43] Ogden A (2017). Multi-institutional study of nuclear KIFC1 as a biomarker of poor prognosis in African American women with triple-negative breast cancer. Sci Rep.

[44] Kalla Singh S (2010). Differential insulin-like growth factor II (IGF-II) expression: a potential role for breast cancer survival disparity. Growth Horm IGF Res.

[45] Lindner R (2013). Molecular phenotypes in triple negative breast cancer from African American patients suggest targets for therapy. PLoS ONE.

[46] Nanda R (2005). Genetic testing in an ethnically diverse cohort of high-risk women: a comparative analysis of BRCA1 and BRCA2 mutations in American families of European and African ancestry. JAMA.

[47] Olopade OI (2003). Breast cancer genetics in African Americans. Cancer.

[48] Haiman CA (2011). A common variant at the TERT-CLPTM1L locus is associated with estrogen receptor-negative breast cancer. Nat Genet.

[49] Ruiz-Narváez EA (2010). Polymorphisms in the TOX3/LOC643714 locus and risk of breast cancer in African-American women. Cancer Epidemiol Biomarkers Prev.

[50] Garlapati C (2019). The persisting puzzle of racial disparity in triple negative breast cancer: looking through a new lens. Front Biosci (Schol Ed).

[51] Deshmukh SK (2017). Emerging evidence for the role of differential tumor microenvironment in breast cancer racial disparity: a closer look at the surroundings. Carcinogenesis.

[52] Yao S et al. Breast tumor microenvironment in black women: a distinct signature of CD8^+^ T cell exhaustion*.* J Natl Cancer Inst. 2021. 10.1093/jnci/djaa215 PMC8328978 33395700

[53] Thorsson V (2018). The immune landscape of cancer. Immunity.

[54] Kinseth MA (2014). Expression differences between African American and Caucasian prostate cancer tissue reveals that stroma is the site of aggressive changes. Int J Cancer.

[55] Wallace TA (2008). Tumor immunobiological differences in prostate cancer between African-American and European-American men. Cancer Res.

[56] Curran T (2021). Differential immune signatures in the tumor microenvironment are associated with colon cancer racial disparities. Cancer Med.

[57] Floris G (2021). Body mass index and tumor-infiltrating lymphocytes in triple-negative breast cancer. J Natl Cancer Inst.

