# Supplementary material for: Quantitative assessment of the immune microenvironment in African American Triple Negative Breast Cancer: a case–control study
Source: Breast Cancer Res. 2021 Dec 14;23:113. doi: 10.1186/s13058-021-01493-w (PMC8670126; doi:10.1186/s13058-021-01493-w)
Supplement: Supplementary file 1 — Additional file 1. Primary antibodies stock concentrations and dilutions. 1Stock concentration not available [file 13058_2021_1493_MOESM1_ESM.docx]

| **ANTIBODY** | **DILUTION** | **STOCK CONCENTRATION** |
| --- | --- | --- |
| CD45 | 1:200 | 375 mg/L |
| CD14 | 1:500 | N/A^1^ |
| CD68 | 1:200 | 40 mg/L |
| CD206 | 1:4000 | 0.569 mg/ml |
| CD8 | 1:250 | 157 mg/L |
| CD4 | 1:400 | 374 mg/L |
| CD3 | 1:100 | N/A^1^ |
| CD20 | 1:150 | 126 mg/L |
| KI67 | 1:100 | 46 mg/L |
| GRANZYME B | 1:2000 | N/A^1^ |
| FOXP3 | 1:50 | N/A^1^ |
| A-SMA | 1:500 | 0.071 mg/ml |
| FAP | 1:500 | 0.683 mg/ml |
| THY1 | 1:10000 | 1 mg/ml |
| COL4 | 1:400 | 1 mg/ml |
| CD34 | 1:4500 | 0.552 mg/ml |
| VWF | 1:50 | N/A^1^ |
| PD-L1 | 1:800 | N/A^1^ |
